# Visioning a food system for equitable transition towards sustainable diets

**DOI:** 10.3390/su14063280

**Published:** 2022-03-10

**Authors:** N. Sobratee, R. Davids, C.B. Chinzila, T. Mabaudhi, P. Scheelbeek, A.T. Modi, A. Dangour, R. Slotow

**Affiliations:** 1 School of Life Sciences, University of KwaZulu-Natal, P. Bag X01, Scottsville 3209, Pietermaritzburg, South Africa; 2 School of Agricultural, Earth and Environmental Sciences, University of KwaZulu-Natal, P. Bag X01, Scottsville 3209, Pietermaritzburg, South Africa; 3 Centre for Transformative Agricultural and Food Systems, School of Agricultural, Earth and Environmental Sciences, University of KwaZulu-Natal, P. Bag X01, Scottsville 3209, Pietermaritzburg, South Africa; 4 London School of Hygiene and Tropical Medicine, London, UK; 5 Centre for Biodiversity and Environment Research, Department of Genetics, Evolution & Environment, University College London, London, UK

**Keywords:** agri-food system, systemic analysis, marginalised communities, sustainable diet, multi-level perspective, deliberative policy-making

## Abstract

The Global Goals to end hunger requires interpretation of problems, and change across multiple domains. We facilitated a workshop aimed at understanding how stakeholders problematise sustainable diet transition (SDT) among a previously-marginalised social group. Using the systems thinking approach, three sub-systems, access to dietary diversity, sustainable beneficiation of natural capital, and ‘food choice for well-being’, highlighted the main forces governing the current context, and future interventions. Moreover, when viewed as co-evolving processes within the multi-level perspective, our identified microlevel leverage points - multi-faceted literacy, youth empowerment, deliberative policy-making, promotion of sustainable diet aspirations - can be linked and developed through existing national macrolevel strategies. Thus, by reconsidering knowledge use in the pursuit sustainability, transformational SDT can streamline multiple outcomes to restructure socio-technical sectors, reconnect people to nature-based solutions and, support legitimate aspirations. The approach could be applied in countries having complex socio-political legacy and to bridge the local-global goals coherently.

## 1 Introduction

The complexity of local and global problems challenge the agricultural, health, and socioeconomic sectors^1,2^. Moreover, the environment and biodiversity are increasingly under threat from climate change and competing development needs^3^. The food system, for instance, both threatens environmental sustainability and nurtures human health^4^. These, and competing societal needs, are addressed within the framework of the United Nations Sustainable Development Goals (SDGs)^5,6^. A major challenge today, and into the future, is to sustain the beneficial contributions of nature^7,8^, whether from natural or managed systems^9^, including food systems^10,11^, to improve wellbeing for all. However, not all countries are able to transition towards equitable development pathways for all, because of slower macroeconomic growth that reduces the pace of structural change in low and middle income countries^4, 12^.

As with its other Sub-Saharan counterparts, South Africa faces multiple biophysical, political, and socioeconomic pressures that interact to compound livelihood vulnerability, and hence limit adaptive capacity^13^. Moreover, the apartheid legacy and delayed transformation suggest that new development strategies and outcomes of institutional arrangements are warranted in tackling socio-economic disparities, such as chronic poverty, household food insecurity^6,14^, and other protracted socio-ecological problems^15^. Well-intended policies can lead to unintended consequences when there are incongruous policies and implementation strategies, such as skewed prioritisation of economic gains over poverty alleviation, local economic development, and/or nature-based food security^16, 17, 12^. Growing evidence and, increasingly, decision-making, focus on developing societal capacity to guide transitions that align with social and environmental alternatives^4,18^. Despite having the potential to promote environmental sustainability while supporting human health and wellbeing,^10,19^ current trends indicate that inequalities will persist^20^.

A broad range of theoretical and conceptual frameworks have been applied to promote transitions towards food sustainability. Herein, we draw insights from the multi-level perspective (MLP) on socio-technical transitions^21, 22^, to better understand how to realise transition towards sustainable diets amongst vulnerable, previously disadvantaged communities in South Africa. The strength of transition research is its ability to address systemic changes through long-term, multi-dimensional, and fundamental transformation processes, towards a more sustainable society^23^, noting that its relevance and applicability within the agri-food sector requires an integrative approach^24,25^. The multi-dimensional concept of sustainability can cause some ambiguity as to the different normative values pertaining to food, and the tension between commodity vs commons, which can be assisted by a more unified worldview amongst diverse stakeholders^26^. Hence, the specific objectives of this paper were to: (i) examine the intricate relationships that emerged when stakeholders collectively interpreted and envisioned conceivable ways to shape “sustainable and healthy food system” as the future desired state, against the state of the current food system; (ii) evaluate, through a scoping review, the concept of transition with respect to agri-food systems; (iii) use a logical framework to demonstrate how the interventions proposed for leveraging sustainable diet transition, call for a consideration of the wider context within which the transition takes place; and (iv) identify contextual pathways to inform future policies guiding sustainable diet transitioning, that take into account the influence of multiple systemic interactions and the type of actors that need to be involved.

## 2 Methods

The present work uses a mixed method approach (Supplementary Material) to co-design emergent research-practice collaboration for the SHEFS programme, a Wellcome Trust (UK)-funded Our Planet Our Health project, in South Africa. We applied systems thinking principles, using causal loop diagramming, to develop insights, make distinctions, i.e., which knowledge disciplines or institutional settings to consider, identify interrelationships and subsystems, and establish the most pertinent perspectives. Through interactive facilitation and mapping, we helped stakeholders to acknowledge and observe the complexity of interventions linked to transdisciplinary sustainability research collaboration. To unpack the complexity issues linked to sustainable and healthy food systems, we aligned the interventions proposed by the workshop participants, viewed as leverage points, together with the SDGs, within a logical framework. Finally, we embedded these leverage points within a multiple level perspective framework, encompassing a niche-regime-landscape continuum^27^, aimed at informing the types of evidence-based policies that could potentially be devised to inform sustainable diet transitioning. The ‘niche-regime-landscape’ multi-level perspective is a prominent framework to analyse socio-technical transitions towards sustainability, which stems from evolutionary economics and social construction of technology^27^. Central to this is that economic processes evolve, and that economic behaviour is determined both by individuals and society as a whole^28^. In the present context, sustainable socio-technical transition, therefore, refers to new kinds of agri-food systems shifts, and the types of actors required to support participatory consensus outcomes that encourage desired change. This work is supported by an ethical approval granted by UKZN, and all workshop participants provided informed consent for their participation.

### 2.1 Systemic analysis of sustainable diet drivers

We captured the outcomes of the first Sustainable and Healthy Food Systems (SHEFS) Programme key stakeholder workshop (30 October 2017, in Durban, South Africa) to define the current and desired state of the agriculture, environment, and social system, in South Africa. The facilitated workshop brought together 39 stakeholders from key government competencies, across the three levels of government policy makers and practitioners (municipality, provincial, and national), and academics and post-graduate students from crop science, food security, nutrition, health sciences, development studies, environmental science, and biodiversity conservation. To facilitate the process, participants were asked to consider, firstly, SHEFS’s (https://shefsglobal.lshtm.ac.uk/) overarching aim: “to provide policy makers with novel, interdisciplinary evidence to define future food systems policies that deliver nutritious and healthy foods in an environmentally sustainable and socially equitable manner” as a guiding star, which is a preferred future state of the system. Secondly, a “near star” question: “What is the effectiveness of the current foodcrop-environment-health system for addressing human livelihoods and welfare, considering knowledge, understanding, legislation, policies, implementation and sustainability?” For this exercise, the workshop participants spent 3 hours in groups, each including representatives of all stakeholder types, to brainstorm and map: (1) the state of knowledge, and (2) the possible desirable states.

We then wanted to capture a systemic overview from each group, through causal diagrams, about how the stakeholders’ mental models related to the SHEFS programme’s overall objectives. Following a briefing on the conventions of drawing interrelationship digraphs (concept terms connected by a bi-directional line)^29^ and causal loop diagrams^30^, the participants in each group were then asked to respond to the questions by drawing their group’s collective interpretation of the system (without idea exchange amongst groups). All diagrams generated were refined by engaging with the participants, through interactive facilitation during the workshop, to ensure that the ideas were accurately captured and representative, and, thereafter, updated by the author team to produce conventional causal loop diagrams (CLDs). CLDs are used to conceptually model dynamic systems, which can be social and/or ecological, by mapping how variables, i.e., factors, issues, processes, influence one another^30, 31^. Common variables that appeared in the different group diagrams were identified and the nature of their causal relationships were highlighted to create the interlinkages among the sub-system, uncover any underlying feedback structures and identify leverage intervention points in the system^32,33^.

The following day, we conducted post-workshop expert deliberations, including the principal SHEFS investigator and nutrition expert (AD), the principal investigators in environment (RS) and crop (AM), the project co-ordinator for South Africa (RS), one researcher in diet and health (PS), and two researchers representing the health sciences co-investigator. Collectively, we acted as key informants to identify science-action interventions, from the previous day’s outcomes, with high leverage impacts for biodiversity (Nature) and end-user beneficiaries (People). During a five-hour focus group discussion, we interrogated the linkages and nature of the different sub-systems identified the previous day, to develop a strategic framing. The causal loop diagrams were reviewed by the experts with the workshop facilitator (NS), and complemented by (i) groundwork that was already being undertaken by the researchers, and, (ii) additional potential research gaps capable of delivering sustainable diet leverages that had not been identified the previous day, but emerged from interrogation of the linkages and causal loops. Causal loop analysis was performed and, where relevant, system archetypes^34^ were applied to present a system view of the interplay between the different forces identified. Colour coding, based on subsystems, identified archetypes and/or inter-linkages, as then used to enhance representation of the diagrams. Relevant literature was used to substantiate, align, unpack the interpretations of the stakeholder views with respect to the guiding star and near star questions.

### 2.2 Review of bibliometric studies on sustainable transition of food systems

#### 2.2.1 Review of multi-level perspective in food agri-systems

The emergence of persistent environmental degradation worldwide has raised the question of how to induce a societal transformation towards more sustainable production, consumption, and biodiversity protection^35^. New technologies or governance approaches, economic deregulation, and changes in consumer behaviour have been introduced to relieve urgent problems^36,37^. However, generally, transformational processes are slow or even failing, technology diffusion is inefficient, governance concepts are executed in theory only, deregulation causes high uncertainties and consumers do not act as anticipated^35^. A broad range of frameworks have been used to explore transition towards sustainability^24^, such as the multi-level perspective (MLP) on socio-technical transitions^38^, transition management^39^ (TM), strategic niche management ^40^ (SNM), technological innovation system^41^ (TIS), and social practice approach^42^ (SPA). The MLP argues that transitions i.e., large-scale socio-technical change, occur through interactions between processes at three levels. First, niche innovations build up impetus, through knowledge production processes such as research and/or performance improvements, and support from powerful civil society groups. Here, the concept of ‘experimentation’ occupies a central position within the academic component that investigate transformations towards sustainable socio-technical systems. The focus on experimentation is a key agent of change that sets the sustainability transitions field apart from the wider literature of social change and policy theory^43 44^. ‘Socio-technical experimentation’ can be contrasted with the notion of experimentation used in the natural sciences. It implies a more engaged and social constructivist position whereby society is itself a laboratory and a variety of real-world actors commit to the messy experimental processes tied up with the introduction of alternative technologies and practices to purposively re-shape social and material realities^43, 45^. Second, the concept of the socio-technical regime has been formulated to account for the delay and path-dependency experienced in articulating and understanding transformative change^46^. Regimes, therefore, results from the co-evolution of institutions and technologies over time that become positioned into practices and routines^47^. Sociologists of technology refer to regimes as consisting of a variety of actors, that is, scientists, policy makers, consumers and special-interest groups that contribute to patterning of technological development^48^. The sociotechnical regime concept therefore, accommodates a broad community of social groups and their alignment of activities and their interactions result in the stabilisation of socio-technical trajectories in many ways: regulations and standards^49^, adaptation of lifestyles to technical systems, investments in machines, infrastructures and competencies^50, 51, 52^. Third, the socio-technical landscape, which could be macro-economics, deep cultural patterns, macro-political developments, constitute an exogenous environment beyond the direct influence of niche and regime features^24^. Changes at the landscape level usually take place over decades, and, such changes can exert pressure on the regime through a selective process of societal change – sectoral policies, education system, market-driven technological novelty - and create ‘windows of opportunity’ for regime change and subsequently providing leverage for niche innovations to emerge and create a new regime^53^. A transition, therefore, occurs when a regime is transformed as it responds to systemic changes. To place things into perspective, then, the purpose of this paper is to interrogate what type of leveraged interventions ought to be in place in order to create a new socio-technical regime wherein sustainable diet becomes dominant. The MLP framework is useful in understanding contexts that have co-evolutionary properties as it aids in justifying the importance for adaptive policy approach when addressing complex problems burdened with intrinsic dynamics^52^.

We conducted a search in the *Web of Science Core Collection* (SCI-EXPANDED, SSCI, A&HC1, ESCI), dated 3 August 2020, for publications on sustainable food transitions, more specifically those that applied the multi-level perspective. The output was narrowed down to include articles that deal with the “food systems” topic. Hence, the search term used was:

TOPIC:(socio-technical transition AND multi-level perspective) AND TOPIC: (food systems)

The search identified 15 articles (n=15), and given the small sample size, all were retained for scrutiny. In the analysis of the output, the following attributes were derived: the context of the transition research, the transition process, any specific of methodologies/approach, and the action domain that emerged.

#### 2.2.2 Developing multi-level insights for sustainable diet transition from the stakeholder systemic analysis

Having explored the MLP transitions in the literature, we then used the output from the stakeholder workshop using the MLP framework, to showcase how evidence-base sustainable diet polices can be rendered more effective in addressing barriers and opportunities, and thereby, to realise sustainability transition in the near future. We identified examples of interventions that were co-designed by stakeholders, and assessed by the expert deliberations, as leverage points within the socio-technical and socio-ecological context. We then categorised those as proposed policy measures against the niche (micro-level) - regime (meso-level) - landscape (meta-level) continuum and described the range of network of actors that can actualise the corresponding transitions.

## 3.0 Results

### 3.1 Impact of extrinsic systemic issues on small-holder farming (SHF) in South Africa

#### 3.1.1 A nation-state level perspective

**Figure 1:**
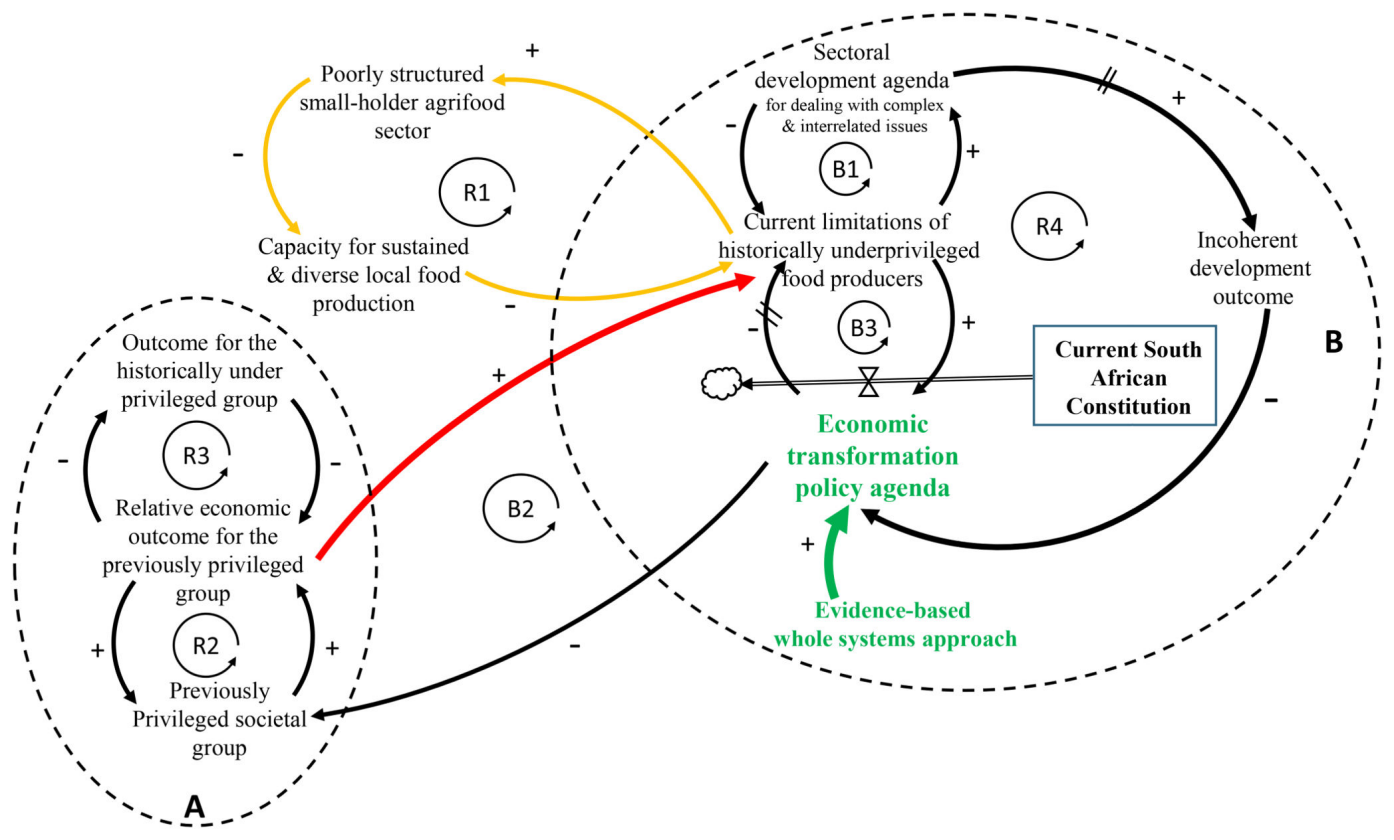
Interlinkages and causation pathways impacting current limitations faced by historically underprivileged smallholder farmers. **A**: A ‘Success to the successful’ archetype that explains South Africa’s previous segregated socio-economic situation; **B**: A ‘Shifting the burden archetype’, whereby an over-emphasis on sectoral development with insufficient implementation of whole systems evidence-based approaches results in incoherent development outcomes that eventually undermine the achievement of the economic transformation. Loop B2 and Red arrow: anomaly that persist for more than two decades among the previously marginalised despite the end of apartheid rule. Ideally, given the inclusive and affirmative opportunities offered through the new Constitution, the current limitations of the historically underprivileged should have decreased with the elimination in white privilege. However, the persistent inequality indicate that this is not the case. This means that development solutions that are being brought about are not tackling the root causes of problems, resulting in inadequate outcomes; e.g., the smallholder agri-food sector remains poorly structured with insufficient capacity for a thriving local production. Loop R1 with yellow arrows: vicious reinforcing consequence that perpetuate limitation in the smallholder food sector. Arrows with double dash: Intrinsic systemic delays characteristics of complex systems; Hour-glass symbol: Dynamic nature of the variable represented as a rate of change, herein indicating that the economic transformation ought to be an on-going adaptive process, driven by the democratic Constitution that act as a guiding principle for a new normal in South African politics to influence governance and steer other multi-dimensional change

Participants interrogated whether the end of apartheid had improved the situation for the South African smallholder farming sector, which is essentially comprised of the previously disadvantaged population. It was agreed that this sector remains seriously limited and poorly structured, being embedded in a reinforcing vicious cycle (R1) (Fig. 1) that undermines capacity-building for sustained and diverse local food production. Not only is the smallholder farming sector disadvantaged from a productivity standpoint, but the institutional dynamics related to the socio-economic conditions render it nearly impossible for smallholder farmers to thrive and emerge as small-scale commercial farmers^54, 55^. Loop B1 (Fig. 1) describes how the economic transformation policy agenda aims to reduce the current limitations of the historically underprivileged smallholder food producers through emphasis on sectoral development planning as is elaborated in the National Development Plan^56^. Under apartheid rule, the relative economic outcome beneficiated the privileged societal group (Loop R2) to the detriment of the historically under-privileged group (Loop R3) (Fig. 1). With the advent of democracy in 1994, the objective of the transformative agenda was to redress privileged beneficiation by opening the economy and progressively offsetting the unequal economic outcomes. In the new South African Constitution ‘every citizen is equally protected by law’ and all are obligated to ‘heal the divisions of the past’ while ‘recognising injustices of the past’ ^57^. Yet, inequality is still pervasive^58^. More than two decades after the dispensation, and despite the various initiatives of the economic transformation processes, the governance and reality of the smallholder farming sector at large still grapples with systemic limitations, as represented in the balancing loop B2 (Fig. 1).

### 3.2 Efficacy of structural adjustment for socio-economic upliftment

#### 3.2.1 Transitioning of smallholder farmers

**Figure 2:**
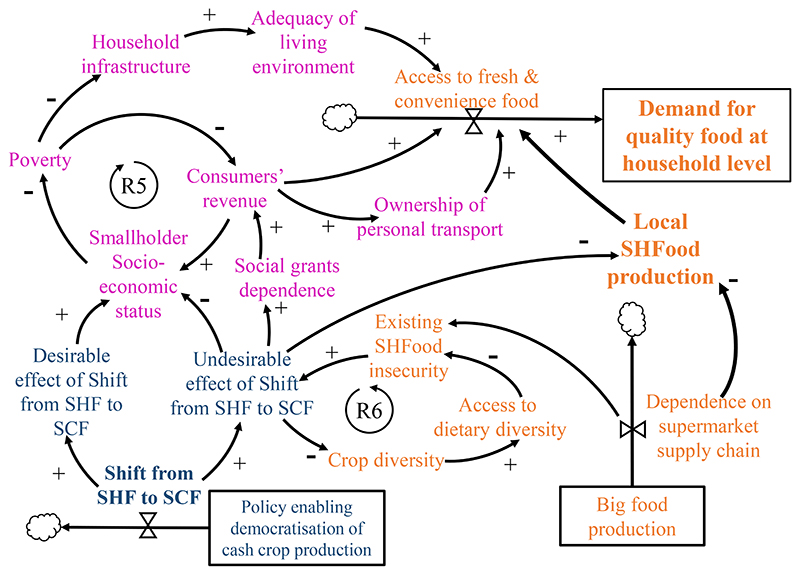
Impact of shift from small-holder farming (SHF) to small-scale commercial farming (SCF) on small-holder socio-economic status. Reinforcing loop R5: The spill-over effects resulted in desirable and undesirable effects on the socio-economic status of the smallholder farmers, that either relieves or exacerbates poverty level depending on how successful they emerge as small-scale commercial farmers. Reinforcing loop R6: Unsuccessful cash crop ventures diminish access to dietary diversity and worsen food insecurity, such that eating habits are linked with key issues around affordability and convenience. Orange variables: Smallholder/previously under-privileged access to food and dietary diversity. Blue variables: effect of policy enabling democratisation of cash crop production. Purple variables: Socio-economic realities of the smallholder sector.

As part of unpacking loop B2 (Fig. 1), and especially the associated causal factors, participants referred to the unintended consequences of agricultural policy. This was based on the premise that creating opportunities for the previously disadvantaged to own and farm land would help subsistence farmers’ attempts at commercialisation, and create a middle group termed the ‘emerging farmer’ sector. Policies enabling the shift from smallholder farming (SHF) to small-scale commercial farming (SCF) has had two types of spill-over effects (Fig. 2). The first one creates a causal pathway with desirable effects whereby the farmers who have enough leverage to invest can improve their socioeconomic status. This would alleviate their poverty level by improving their flow of household revenue (Loop R5). Subsequently, they can improve their living standard, and, in effect, ensure access fresh and convenience foods. The variable ‘consumer’s revenue’ here refers to the previously disadvantaged population that are also food consumers in the SHF system.

The second effect is when the farmers, despite aspiring to farm successfully, still find their socio-economic and household food security status undermined. This occurs due to a combination of factors ^59, 60^such as poor business framework and insufficient input support, know-how, and infrastructure, hence, contributing to the undesirable effect on the shift from SHF to SCF. The example of cash crop production, such as sugarcane in the KwaZulu Natal Province, was used to illustrate the unintended consequence in the reinforcing vicious loop, R6. In striving to produce sugarcane as a monocrop, on-farm crop diversity is reduced because food crops are neglected. Dietary diversity within such households, which depends on subsistence farming, is undermined, leading to household food and nutrition insecurity. These consumers must increasingly rely on the ‘Big Food Industry’. Such type of food sourcing from supermarket outlets creates a dependence on supermarket supply chains, which is unaffordable and inaccessible to poor communities, further exacerbating existing household food insecurity.

#### 3.2.2 Impact of socio-economic conditions on access to healthy and sustainable diet

When the socio-economic status of smallholder and underprivileged communities result in sub-optimal revenue, poverty remains rampant and pervasive. Ubiquitous prevalence of poverty creates dependence on social grants to support household revenue for consumption. This dependence is counter to other policy decisions, such as improving smallholder socio-economic status through economically sustainable means. The ‘adequacy of the living environment’, itself dependent on revenue generation, is a critical factor that prescribes the type of food consumed (Figure 2). The poor and previously underprivileged communities occur in the peri-urban region as sub-organised settlements or as informal segments in the metropolitan cities. It is only when adequate revenue is allocated towards household infrastructure and facilities, such as access to electricity and ability to store perishable and/or convenience food in a refrigerator, and ownership of car or access to other form of transport, that access to food can be definite at the household level.

**Figure 3:**
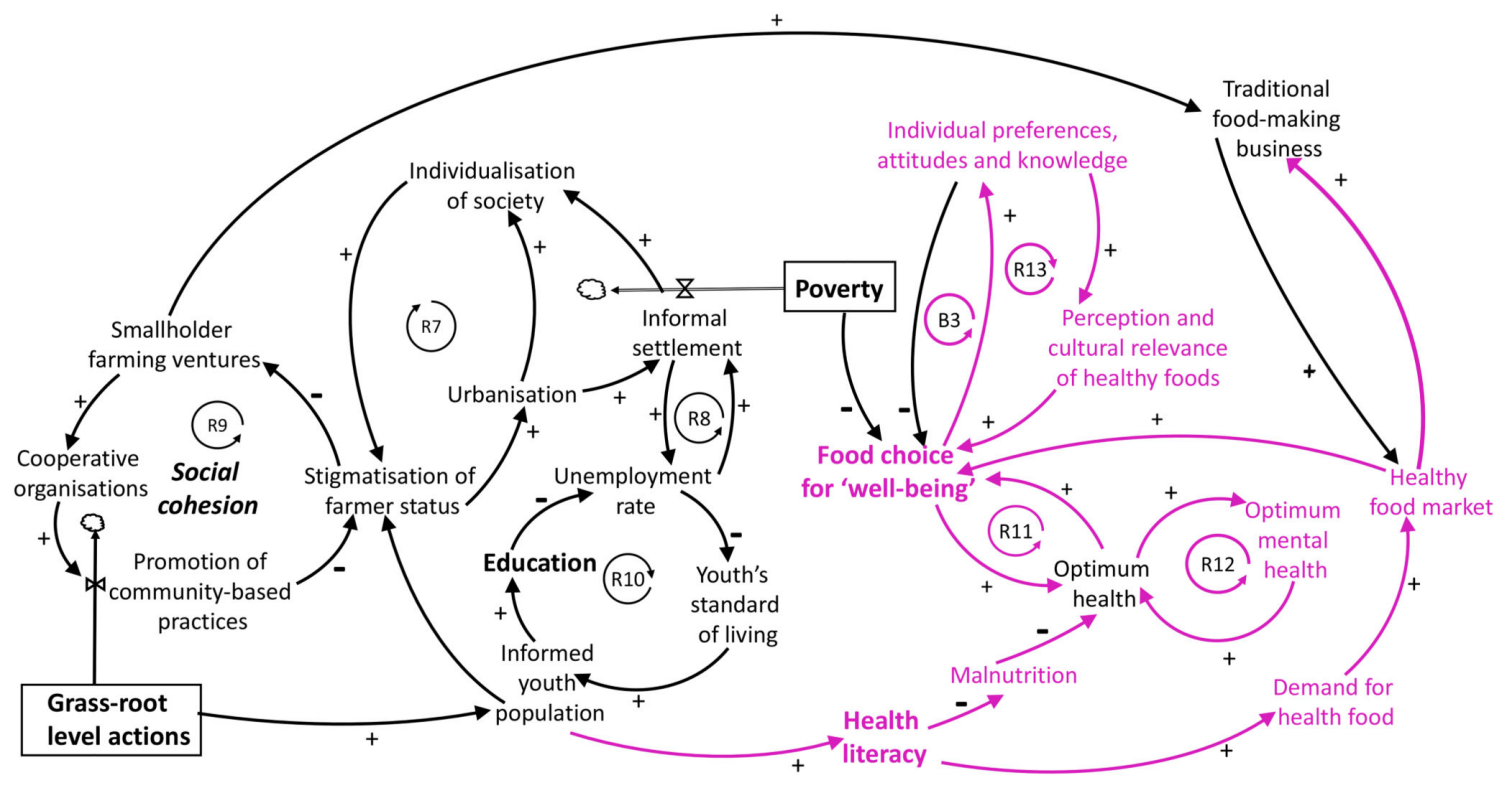
Socioeconomic factors that impact small-holder farming ventures and ‘Food choice for wellbeing’. Blue variables: Spill-over effect of de-agrarisation through stigmatisation leading to proliferation of informal settlement and unemployment. Pink variables: Influence of ‘health literacy’ in leveraging food aspirations and ‘food choice for well-being’. Stimulating small holder farming ventures and driving the demand for healthy food market ought to stimulate traditional food-making business which could then influence a positive feedback upon food aspirations and choice. ‘Traditional food making business’ emerged as a currency to stimulate both smallholder farming ventures and to create a drive for healthy food market and eventually ‘Food choice for well-being’.

Moreover, participants referred to the fact that the ways the previously under-privileged people consume food culturally, and the historically-conditioned meanings ascribed to food and eating, have be considered in order to understand how to shift current food consumption towards sustainable transition. The emerging patterns^61^ consists of a preference for cheap grain staples, sugar, soft drinks, and chicken frequently sourced through informal channels. This implies that, apart from price and convenience, the symbolic and aspirational domain of food aesthetics and the social functions of visible consumption become key forces shaping the food choice. Currently, individual preferences and attitudes are stronger determinants of food choice, rather than sustainable food choice for well-being acting as determinants of food choice(Fig. 3). This is a consequence of the increasing individualisation of society, an outcome of western lifestyle fast-food aspirations. When it comes to food choice and consumption, on the one hand, the individualisation of lifestyle and lavish food preferences represent the fulfilment of historically unfulfilled desires dating from apartheid rule. The resulting attitude renders ‘past foods’, mainly maize porridge and vegetables, as undesirable reminiscence of the ‘difficult past’, and healthy food is perceived as unappealing or too expensive. On the other hand, the sprawl of informal settlements and abject poverty lead to poor food choice due to financial constraints and inability to afford healthy food [Poverty → Food choice for well-being (Fig. 3)]. Both situations are not aligned with food choices that promote well-being.

The individualisation of lifestyle is the outcome of a spill-over effect resulting from a vicious reinforcing loop involving stigmatisation of farmer status and urbanisation, as seen in R7 (Fig. 3). Participants discussed how, despite the political will for a more inclusive agricultural economy, smallholder farming has been on the decline in recent years because of a combination of macro-economic constraints. In particular, the stigmatisation of farming activities has discouraged youth participation in agriculture ^62, 63^. Post-apartheid de-incentivisation of agriculture was deemed as the major systemic barrier that deterred communities from sustaining small-holder farming. Coming from a difficult past characterised by restrictions on movement, education, wealth accumulation, among other things, the palpable post-apartheid response has seen an increase in movement, leading to the rural exodus because of the perceived opportunities and prosperity that the urban regions could potentially provide.

### 3.3 Interventions to leverage sustainable diet transition

#### 3.3.1 Socio-economic factors, social aspirations, and individual food choice behaviour

Participants posited that high leverage interventions would necessarily have to include improving health literacy of consumers to tackle problems of malnutrition, and to create the demand for healthy food. Therefore, instead of “Individual preferences, attitudes and knowledge” influencing whether consumers opt for “Food choice for well-being”, participants proposed that sustainable diet transition should be stimulated in such a way that “Food choice for well-being” becomes the determinant for food preferences and attitudes. As such, individual choice is a complex dietary behaviour and is influenced by various physiological, social and cultural factors^64,65^. In Figure 3, this is represented as the balancing loop, B3. As a result of the “Food choice for well-being” → “Individual preferences, attitudes and knowledge” relationship, a desirable and aspired loop is created as R13.

Table 1 explains the causation pathway from the proposed interventions to expected outcomes. The UN SDGs are used to provide the overarching context and relevance of the transformative trajectory.

#### 3.3.2 Reinforcing the democratisation of knowledge to unleash sustainable diet transitions

**Figure 4:**
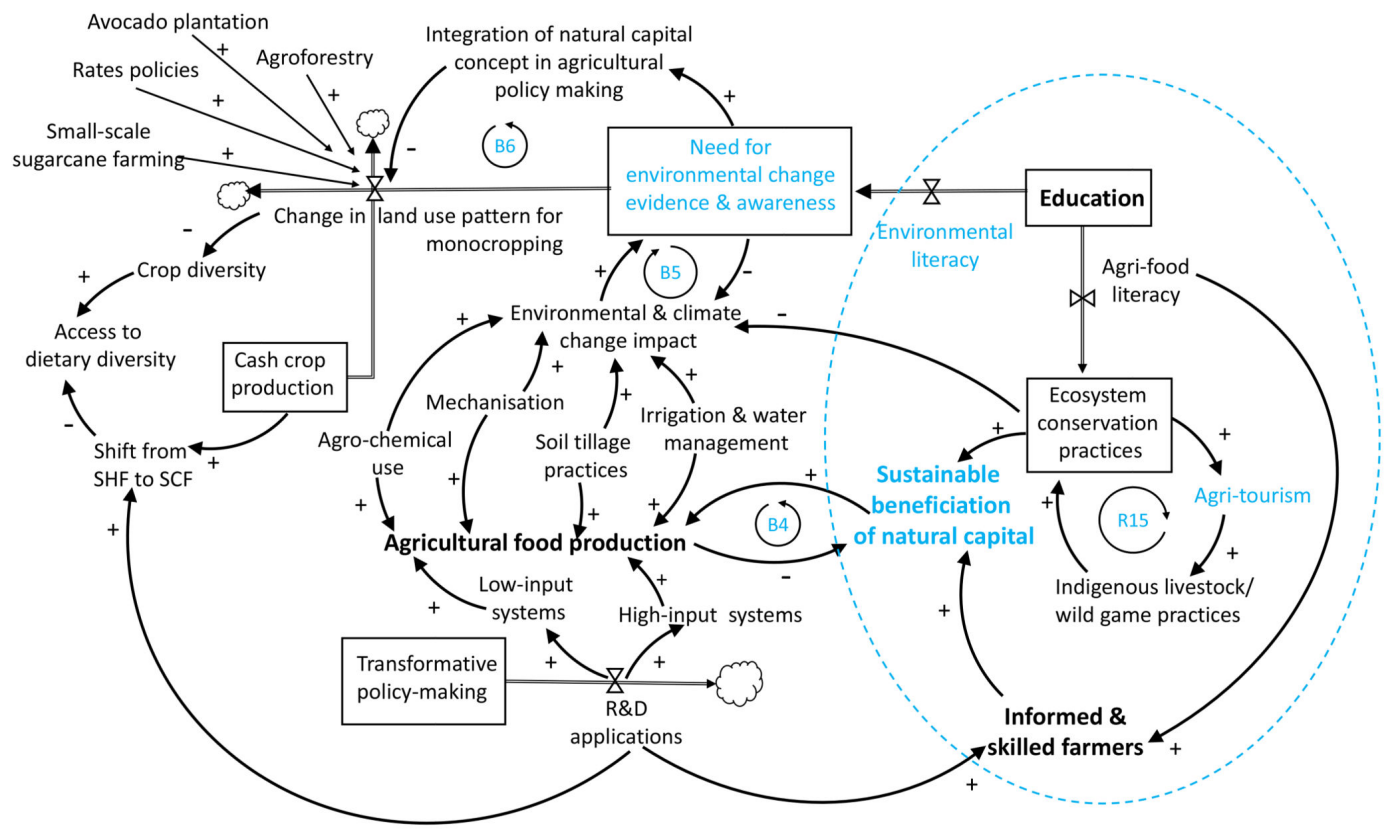
Emergence of functional education as a key nexus to improve environmental awareness and promote agri-food literacy. Agricultural food production in South Africa is essentially a dual system consisting of the low-input subsistence system and the high-input commercial systems that are stimulated through on-going R&D policies. Agricultural operations (mechanisation, irrigation and water management, soil tillage practices) and input (agro-chemical use) applied to boost food production worsen climate change and environmental impacts and have a dampening effect on the sustainable beneficiation of natural capital (Balancing Loop B4). Emphasis on multi-faceted literacy ought to incentivise social entrepreneurial innovation and sustainable beneficiation of natural capital from agricultural activities (Loop R15: virtuous reinforcing loop where the variables mutually reinforce agritourism, indigenous practices and promote ecosystem conservation practices; all stimulated through functional agri-literacy). Loop B5: Evidence-building and awareness can reduce the impact of agricultural activities. Loop B6: On-going evidence-building and creation of awareness regarding environmental change ought to influence multi-sectoral policymaking, for instance by framing natural capital as transformational

Based on the types of interventions endorsed by the participants, the theme of education emerged as a common enabling concept in addressing the limitations of the smallholder sector with respect to sustainable diet transitions and environmental conservation. Functional education could leverage the implementation of sustainable income-generating community-based interventions to promote food security, and sustainable beneficiation of natural capital from agriculture and related novel entrepreneurial activities. Participants referred to the Strategic Plan for South African Agriculture^66^, dated as far back as 2001, which has already aimed to increase incomes of the poorest groups in society through opportunities for small/medium-scale farmers. In effect, the National Department of Agriculture^61^ gives particular attention to small-scale agriculture with three strategic aims: (a) making the sector more efficient and internationally competitive, (b) supporting production and stimulating an increase in the number of new small-scale and medium-scale farmers, and (c) conserving agricultural natural resources. However, these aims are yet to gain adequate leverage^67^, and, hence, are still relevant as expected outcomes of multi-lateral evidence-based interventions in achieving sustainable and healthy food systems. Environmental literacy and agri-food literacy were deemed as important drivers to leverage new types of ecosystem services through inclusive social innovation (Figure 4). The example of reduced crop diversity as an outcome of sector-based thinking in policy planning was mentioned again by participants. In this instance the lens of coherence in land use planning was used to explain how change in land use patterns (Linkage between ‘Change in land use pattern for monocropping’ → ‘Crop diversity’, Figure 4), caused by avocado, sugarcane, and agroforestry, when unchecked, can jeopardise crop and food plate diversity. Therefore, on-going evidence synthesis on environmental change (Loop B5) in local sustainability experiments would be important in understanding how to alleviate cross-cutting issues and unintended consequences arising from sectoral policy decisions.

Ideally, the democratisation of knowledge ought to strengthen bottom-up actions, e.g. in the form of cooperative organisations, civic actions, to deliver greater awareness of policy incentives to community members. Moreover, the inclusion of curriculum and governance components that enable the formalisation of Indigenous Knowledge System (IKS) ought to complement mainstream education, to enhance the on-going development of the much-aspired knowledge-based economy (Loop R16). Participants emphasised that to provide a consolidated frame of action to such an endeavour would require the inclusion of a vibrant policy process that is designed to be adaptive in accommodating IKS (Loop 17). An improved organisation of democracy and civic interest could create sufficient grounds to render the education system more contextually functional, improve employment relevance for the youth and, consequently, their standard of living. The ability to make informed choice would further motivate the pursuit for appropriate information, and enhance the subjective appropriation of their own life course based on sustainable well-being tenets, amongst others. Such a course of action would enable youth transitioning into responsible citizenship. Young individuals will have garnered better understanding of individual responsibility with respect to the different dimensions of sustainable well-being, for instance in terms of diet and health choices, and the shaping of environmental civic engagement. Table 2 displays the transformative pathways capable of leveraging sustainable and equitable food security from a knowledge economy perspective. It shows, amongst others, the comparative advantage of including IKS in policies. This could become an opportunity to adjust the general concept of innovation system to local contexts and practices, and include bottom-up socio-ecological approaches to create stimulus for biodiversity and conservation-friendly entrepreneurial and social innovation. The expected outcomes would have direct relevance to several UN SDGs as shown in Table 2.[Fig F5]

### 3.4 Mobilising systems and coalition of actors for sustainable diet transition

#### 3.4.1 Review of food systems sustainability transition

The use of transition systems research in agri-food systems^24,68,69^ becomes prominent when the problem is complex, ambiguous, and requires the concerted action of many different types of actors to make transformation processes effective. The dialectic relationship between stability (i.e. established rules, governance, habits) versus desired and feasible change in understanding how transition occurs, is central. There are multiple interpretations of what is to be sustained and what is to be developed when considering any particular socio-technical system. This is because there are multiple goals and pathways for development, but in practice, only a subset will be fully pursued. Knowledge is also socially-constructed and politics of power influence explain why some systems or certain sustainability goals tend to be prioritised. In the MLP framing, the concept of “local sustainability experiments” is used to describe what would be the sectors and actors co-existing and operating at the niche level to create novelty. When the unit of analysis lies on sociotechnical systems, the analysis involves a wide range of actors, and no agent has full accountability nor ownership of sociotechnical systems. The novelties can be a combination of scientific research or civil society actions that generate evidence for change.

In agri-food systems, the multiple level perspective is useful to empower communities to generate grass-root and social innovations^70^. As such, it is a long-term process, spanning decades, and characterised by uncertainty and open-endedness. In effect, sustainability journeys are intrinsically dynamic as there are multiple transition pathways, which implies multiple values, and disagreement, since the sustainability notion is highly contested^71^. To catalyse desirable changes in such context, public policy^72,73^ plays a central role in shaping the sustainability transition. As a means to support evidence based understanding of transition transformation whereby the different dimensions of socio-technical systems transitions are considered, various research constructs are used as methods and/or approaches^74^ such as systems thinking^75^, system diagnosis^76^, retroduction^74^, scenario analysis^77^, critical realism are applied to design the interdisciplinary space that requires action.

#### 3.4.2 Empowering vulnerable communities to achieve sustainable diet pathways

Table 3 illustrates how the leverage points can be developed to generate evidence capable of stimulating the policy making process. Based on the multi-level perspective of the socio-technical transitions, the proposed leverages are expressed as policy measures that could be developed, and the categories of actors that could influence the cross-scale transformation process identified. Thus, using the reference of the overarching objective of the SHEFS programme, society is viewed as a set of overlapping socio-technical systems consisting of networks of actors such as consumers, environmental action partnerships, small-scale food producers/farmers, socio-cultural/ non-governmental organisations, value chain financing specialist, and youth/women groups, who act upon institutions, cultural practices, knowledge. Much emphasis is laid on developing substantiative equality, given the socio-political legacy of South Africa. At the niche levels this can be achieved through local experiments on agri-food systems, not only as a science but to unleash capabilities, empowerment, inclusivity, and embracing the socio-ecological viewpoint. For instance, at the time of conducting the current workshop, the Neglected and Underutilised Species (NUS) component of the project had started to generate evidence through scoping reviews and multi-criteria suitability analysis, which subsequently informed a policy brief^78, 79,80,81^.

Because agents/stakeholders with different behavioural characteristics play a role in the distinct stages of transitions, notably pre-development, take-off, acceleration, and stabilisation (establishing the change over time)^82^, they influence the transition process through their goals, knowledge, information, power, interactions, relations, and interests. Thus, for instance, regime-level policy measures that need to be designed to advance rural agrotourism as a development tool must consider new transformational challenges. For agro-tourism to exist not only requires mastering ecosystem conservation and indigenous wildlife practices, but entails a seamless harmonisation with rural entrepreneurship processes in order to become transformational transitions^83,84^. Criterion 8 of the IUCN standard emphasizes the need to learn from the implementation of nature-based solutions (Nbs) to ‘trigger transformative change’ ^85^. However, for this to be realised, NbS must be framed as transformational. The framing of an issue is a key point of focus in transformations, as it influences how people understand the topic itself, shaping how problems and solutions are defined and addressed^86,87^. To catalyse change, the drive for successful transition can be addressed by beginning with developing policies with positive reinforcing loops between the niche (micro-level triggers) and the window of opportunities provided at the landscape (macro) levels.

## 4 Discussion

### 4.1 Understanding the mechanism used to co-design change towards sustainable diets

The study uses an interactive facilitation process among stakeholders to envision and co-design a future state of the food system by prioritising the research focus for the SHEFS consortium that ought to be both sustainable and healthy for the smallholder system and previously disadvantaged group in South Africa. To this end, policymaking would require interdisciplinary evidence capable of leveraging the outcomes of future implementation efforts. We have shown that due to the inherently complex nature of socio-technical and socio-ecological systems within which the notion of sustainable diets have to be embedded, most intervention strategies are likely take effect by way of multiple mechanisms (Intervention → Causal pathway → Expected outcomes → Relevance to global goals) - although it remains an empirical and/or contextual issue whether one mechanism is primary, and others are ancillary. In effect, it is also likely that the same mechanism might be involved in the operation of multiple implementation strategies as shown in the causal loop diagrams (CLDs). To gain clarity on the emergent outcomes of the CLDs, these were unpacked in a logical framework. After having defined the interventions and acknowledging that these processes take place in a multi-dimensional space – comprising institutional rules, economic requirements, multi-level political negotiations as well as social and cultural rules and expectations – we explain the causal pathways in order to improve clarity on the relevance of the proposed corresponding mechanisms that ought to produce the expected outcomes. Herein, following Lewis et al.^88^, we consider “mechanisms” as the processes or events through which an implementation strategy functions to achieve desired outcomes. Careful considerations were taken to ensure that strategic interventions are well-specified and judiciously linked to corresponding mechanisms in a coherent manner. This is because underspecified strategies can potentially leave the interdisciplinary research space vulnerable to inappropriately synthesising data across studies^89,90^. It is to ensure such coherence that the interventions proposed by the stakeholders during the workshop were derived through causal pathways and system loops.

### 4.2 Emergent entry-points for transformative evidence building

Five inter-linked areas have emerged from the stakeholder engagement process which can be used to define priority entry points to build evidence-based policies that align with sustainable and healthy food systems. The first one refers to breaking the legacy of apartheid by advocating transformative governance that acknowledges the pervasive disconnect between, on the one hand, the microlevel socio-political reality of the previously disadvantaged, parochial evidence synthesis and practice and, on the other hand, the positive expectations of the macro-level landscape - Bill of Rights in the South African Constitution^91, 92^ - but which is crippled with counterintuitive effects due to emphasis on sectoral development agenda that result in decade-long pervasive delays to alleviate the smallholder sector. Successful political transformation, that is the shift to democratic South Africa, has not realistically ensued a new normal in terms of social and economic transformation, especially for historically underprivileged smallholder food producers, as the country remains the most unequal society^93^. Second, there was consensus that the critical challenges to be acknowledged in realising intervention efforts requires multi-dimensional evidence-based policy solutions that exhibit dynamics of three functional properties of a knowledge economy in relation to wider transformative processes: identifying positive feedback patterns through education to accumulate multi-functional capabilities, nurturing evidence synthesis for improved practice by way of informational and adaptive policymaking, and empowerment of youth through grass-root actions to capacitate social cohesion, dignity and identity construction. Third, the development and governance of the smallholder food sector ought to foster environmentally sustainable and resilient food systems that can mitigate the impact of unintended consequences of policies that promote commercialisation/intensification of food production to the detriment of subsistence farming, household food security, foodcrop diversity and dietary diversity. Fourth, the ensuing dietary diversity could be partly aligned with the needs of providing healthy diets. And, to further essential nutrition actions, supportive educational measures promoted by health literacy ought to guide social ambitions towards food choice for well-being by promoting sustainable and healthy behavioural shift when aspiring to transition from traditional to modern lifestyle. Fifth, proper recognition of the importance of environmental literacy by mainstreaming awareness of biodiversity loss and its negatively reinforcing impacts across socio-ecological systems. At the same time, environmental literacy could improve cross-sectoral evidence on natural capital sustainability and support the expansion of entirely novel sectors such as agri-tourism at the smallholder level.

The process used for the interactive facilitation and subsequently embedding the emergent outcomes in the MLP of the transition systems framework has contributed to a broader reflection on deliberatively strategising, shaping and modulating sustainable diet pathways towards desirable individual and societal outcomes in full awareness of the scale, influence and urgency of the effort required.

## 5.0 Conclusions

The emergent outcomes of the current work demonstrate the complex nature of sustainable diet by highlighting the multiple interdependencies across sectors and cross-scale dynamics. Intervention strategies to inform policies, therefore, cannot be designed to be stand-alone approaches. Rather, emphasis should be laid on co-evolutionary sets of measures because reliance on generating evidence parochially is not sufficient to inform decision-making for the real world. This work examines key issues raised by stakeholders’ considerations by combining causal mechanisms leading to sustainable diets and embedding the proposed strategies in a multi-level perspective of the transition theory. The mapping of these issues builds knowledge from, and for, practice, by linking different perspectives, including dietary diversity, sustainable beneficiation of natural capital, and food choice for well-being. We have set out five major emergent outcomes of the co-designing process with stakeholders. Despite the very wide knowledge base, disciplines and methodological differences involved in framing sustainable diets in South Africa, the application of causal mechanisms provide a robust grounds for a common framing of analytical and policy-making problems that could be addressed by combining different lenses, styles of explanation and contexts; resulting in the development of substantial bodies of empirical evidence to inform policymaking.

## Supplementary Material

Supplementary Materials

## Figures and Tables

**Figure 5 F5:**
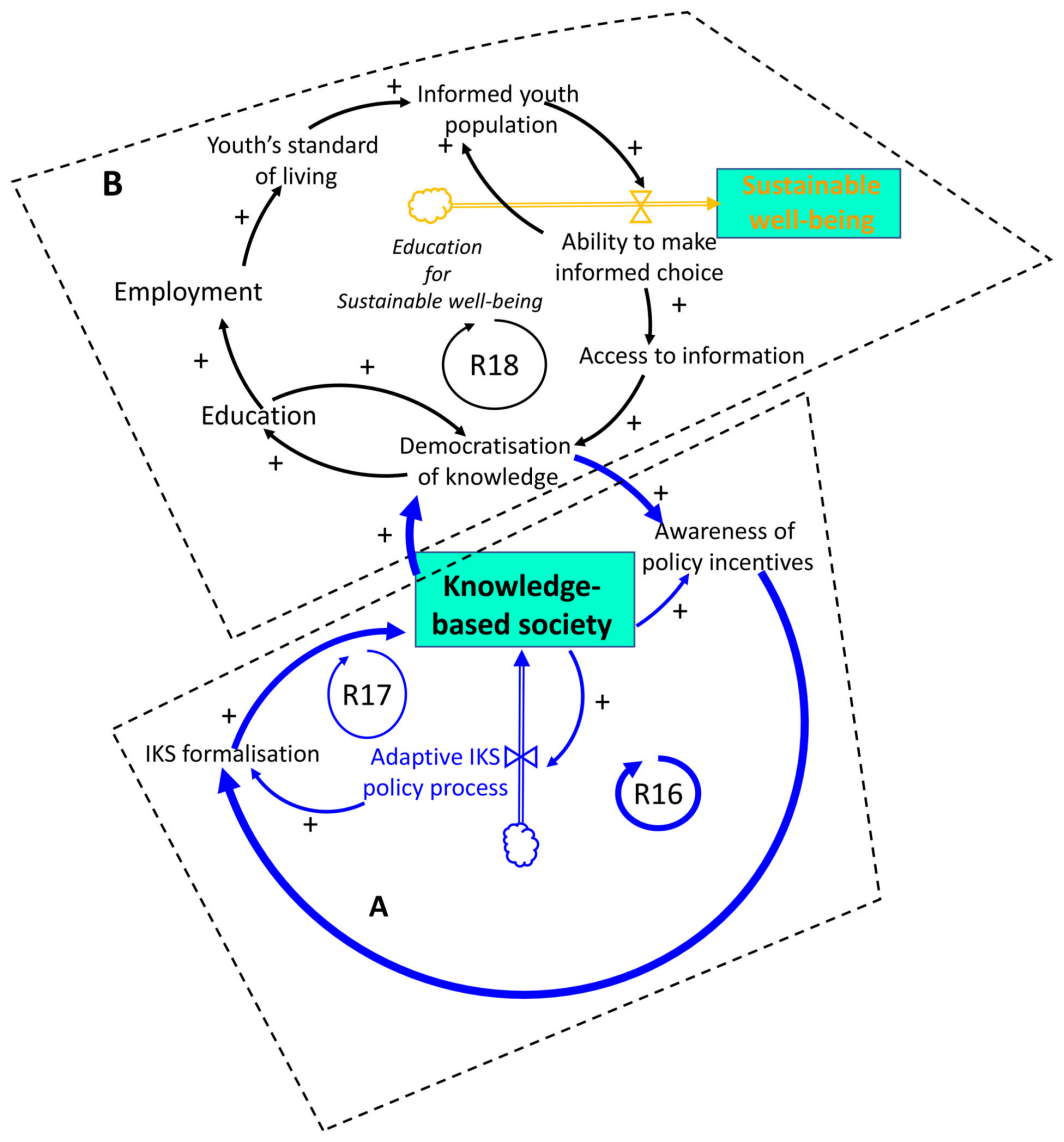
Interventions capable of driving sustainable well-being from the education perspective. **A**: Reinforcing loop, R16, on the consolidation of knowledge-based society by inclusion of Indigenous Knowledge System (IKS) formalisation, mediated by the democratisation of knowledge and awareness of policy incentives. Loop R17: An adaptive IKS policy process reinforces the inclusion of indigenous cultural capital and knowledge; **B**: Tackling unintended effects of social exclusion of youth by using education as a mechanism to enable and drive responsible individual choice for sustainable well-being.

**Table 1:** Transformative pathways to influence food related social aspirations towards sustainable and healthy food pathways. Loops and variables unpacked are from the causal loop diagram in Figure 3. Causal pathways are relationships that are anticipated to generate expected outcomes; Impacts of interventions could occur through different pathways but eventually share the same overarching sets of UNSDG outcomes. The relevant United Sustainable Development Goals (UNSDGs) targets are from: GOAL 2: Zero Hunger; GOAL 3: Good health and well-being; GOAL 4: Quality Education; GOAL 10: Reduced Inequality; GOAL 12: Responsible Consumption and Production.

Interventions	Causal pathway	Expected Outcomes	Relevance as functionally interrelated SDG Targets
Socio-economic factors, social aspirations, and individual food choice behaviour			
Mobilise cross-sectoral resources to promote sustainable diet choices through health literacy	Health literacy→ R11: Pathway to influence food choice that promote health and well-being	Health literacy to reduce malnutrition and improve health, including mental health	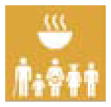 *T2.2* End all forms of malnutrition
			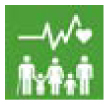 *T3.4* Reduce mortality from non-communicable diseases and promote mental health
	R12: A reinforcing loop that highlights the holistic nature of health as comprising of both physiological health and mental health	Diet and lifestyle based on “Food choice for well-being”	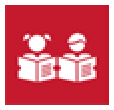 *T4.6* Universal literacy and numeracy
			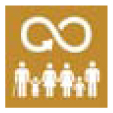 *T12.8* Promote universal understanding of sustainable lifestyle
Support growing traditional and healthy food-making	Spill-over effects of boosting small-scale farm ventures to promote healthy traditional food-making	Driving consumer demand to create a market for healthy local food and support agri-food entrepreneurship	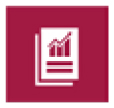 *T8.3* Promote policies to support job creation and growing enterprises
Foster pro-poor food choice for high-quality sustainable diets	R13: A desirable and aspired reinforcing loop which only occur if ‘food choice for well-being’ can influence ‘Individual preferences & attitudes’	‘Food choice for well-being’ habit positively impact ‘Individual preferences and attitudes’, which can then lever ‘Perception & cultural relevance of healthy foods’	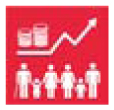 *T1.1* Eradicate extreme poverty food
			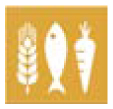 *T 2.1* Universal access to safe and nutritious
			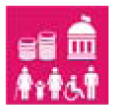 *T 10.2* Promote universal socio-economic and political inclusion
	B3: An important goal-seeking loop to improve preferences & attitudes which cannot be achieved without the ‘health literacy’ causal pathway and outcome of loop R11, to then, link Food choice for well-being→ Individual preferences & attitudes. B3 is however compounded by poverty level.	Successful change in behaviour, provided food choice determinant such as poverty level and therefore access to food, are tackled. A pro-poor sustainable lifestyle would counteract individual preferences and attitudes which do not align with healthy diet pathways	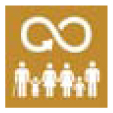 *T12.8* Promote universal understanding of sustainable lifestyle

**Table 2:** Transformative pathways to leverage sustainable and equitable food security from a knowledge economy perspective. Loops and variables unpacked are from the causal loop diagrams in Figures 3, 4 4, and 5. The interventions and their impact indicated through causal relationship(s) are described. The outcomes created for successful transition towards sustainable diet transition are shown with the relevant United Sustainable Development Goals Targets (https://www.globalgoals.org/resources). Main goals are - GOAL 1: No Poverty; GOAL 2: Zero Hunger; GOAL 4: Quality Education; GOAL 8: Decent Work and Economic Growth; GOAL 9: Industry, Innovation and Infrastructure; GOAL 10: Reduced Inequality; GOAL 13: Climate Action; Goal 15: Life on Land; GOAL 16: Peace and Justice Strong Institutions. Causal pathways are thought to generate expected outcomes; Impacts of interventions have different pathways but can have the same overarching sets of outcomes as per the SDG Targets; LED: Local Economic Development

Interventions	Causal pathway	Expected Outcomes	Relevance as functionally interrelated SDG Targets	
Implementation of sustainable income-generating community-based interventions to promote food security and alleviate poverty for the marginalised within a knowledge economy perspective				
Education as vehicles for sustainable development actions	Environmental & Agri-food Literacy with Loop R15: Reinforcing virtuous loop where agri-food and environmental literacy could leverage the development of joint entrepreneurial ventures to boost indigenous livestock & wild game practices	Use capabilities of functional education to create stimulus for biodiversity and conservation-friendly entrepreneurial and social innovation Uplift, promote and preserve indigenous conservation practices and know-how Sustainable use of ecosystem services as innovation instruments to reduce social inequality	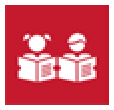	T4.6 Universal literacy and numeracy
			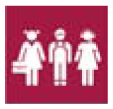	T8.6 Promote youth employment, education and training
			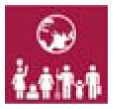	T8.9 Promote beneficial and sustainable tourism
			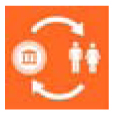	T9.3 Increase access to financial services and markets
			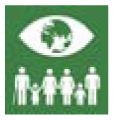	T13.3 Build knowledge and capacity to meet climate change
			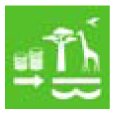	T15.A Increase financial resources to conserve and sustainably use ecosystems and biodiversity
	R18: ’Education for sustainable well-being’ to elevate the youth’s standard of living and knowledge base	An educated youth would cultivate the capacity of discernment for: satisfaction with sustainable lifestyle and built environment employability & entrepreneurial opportunities awareness adequate access to information in order to be informed on food choice and guide individual aspirations	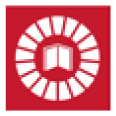	*T4.7* Education for sustainable development and global citizenship
			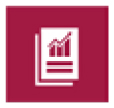	*T8.3* Promote policies to support job creation and growing enterprises
			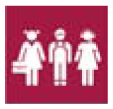	T8.6 Promote youth employment, education and training
			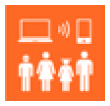	T9.C Universal access to information and communications technology
			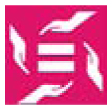	*T10.3* Ensure equal opportunities and end discrimination
			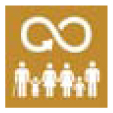	*T12.8* Promote universal understanding of sustainable lifestyle
Promotion of informational governance	R9: Reinforcing virtuous loop aimed at strengthening collective actions through cooperative and social organisations to promote LED	Strengthening of social cohesion through grass-root level actions Capacitate dignity and identity construction to advocate a novel idea around the status of rural and/or peri-urban farming	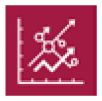	T8.2 Diversify, innovate and upgrade for economic productivity
			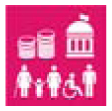	T10.2 Promote universal social, economic and political inclusion
			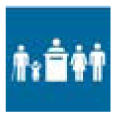	T16.7 Ensure responsive, inclusive, participatory and representative decision-making at all levels
	R16: IKS based policies to improve implementation coherence in a knowledge-based economy	Creation of IKS-based comparative advantages and contextual rationale for positive societal change in the previously marginalised communities	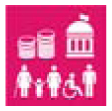	*T10.2* Promote universal socio-economic and political inclusion
			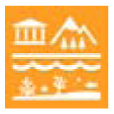	*T11.3* Protect the world’s cultural and natural heritage
	R17: Raising awareness of the advantages of policy incentives ought to boost the formalisation of IKS through an adaptive process		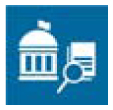	*T16.6* Develop effective, accountable and transparent institutions at all levels
			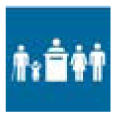	T16.7 Ensure responsive, inclusive, participatory and representative decision-making at all levels

**Table 3:** Multi-dimensional and multi-scalar interactions among the sustainable diet transition sectors, technology, markets, policy and culture, capturing the complexity of systematic changes towards sustainability. NUS: Neglected and Underutilised Species. IKS: Indigenous Knowledge Systems

Level	Policy measure	Example of interventions that can leverage the notion of sustainable diet within socio-technical and socio-ecological systems	Stakeholders as Coalition of actors					
			Consumers	Environmental action partnerships	Producers/farmers	Socio-cultural NGOs	Value chain financing specialist	Youth/Women groups
Niche Micro level: Stimulation of local experiments refers to inclusive, practice-based and challenge-led socio-technical initiative designed to promote system innovation through social learning under conditions of uncertainty and ambiguity	Policies supporting niches	Elaborating effective schemes for embarking in: NUS crop production value chain Promotion of crop and dietary diversity	✓		✓		✓	✓
	Support for the creation of niche networks between various stakeholders	Establishing communication channels between stakeholders: 1. Fostering access to credit/value chain establishment at small-scale levels	✓	✓	✓		✓	
		2. Mainstreaming awareness of biodiversity loss and the cascading impacts across socio-ecological systems	✓	✓	✓			
	Monitoring food choice determinants	Understanding shift from traditional to modernity through lived experience Social media analysis of food choice	✓		✓	✓		✓
		Public co-funding of bottom-up initiatives: small-scale traditional (Gogo, meaning Grandmother) food canteens	✓	✓	✓	✓	✓	
	Normalising environmental impacts of land use shifts	Systematic mapping of sugarcane and forestry land use			✓			
	Supervision of sustainable beneficiation of natural capital	Improve cross-sectoral evidence on natural capital sustainability		✓	✓			✓
**Regime** Mesolevel: The Food Environment that needs to be changed, but consist of dominant actors, institutions, practices, and presumed shared objectives	Support for the expansion of a targeted sector	Rural agro-tourism	✓	✓	✓	✓	✓	
		Education	✓	✓	✓	✓		✓
	Policies limiting the power of regimes	Transparency of lobbying processes	✓	✓	✓	✓		✓
		IKS inclusion						
	Promotion of technical or resource diversity	Public R&D investments and subsidising private R&D in agroecological intensification		✓	✓			
	Regulating unhealthy consumption activities	Taxes or tradable permits, command-and-control of products Sugar tax, fast food	✓		✓			
**Landscape** Meta level Economic, ecological, socio-political, conditions, e.g., South African Constitution that provide the context to drive niche experiments and actions	Promotion of civic debate	Public participation in policy development (round tables).	✓	✓	✓	✓		✓
	Information provision	Informative campaigns for consumer behaviour	✓					✓
	Creation of informed debate	Supporting public participation in setting the policy agenda	✓	✓	✓	✓		✓
	Developing policy integration (technology, environment, consumers)	Making one ministry responsible for coordinating all initiatives and policies concerning long term sustainability transition	✓	✓	✓			
