# Supplementary material for: Visioning a food system for equitable transition towards sustainable diets
Source: Sustainability. Author manuscript; Available in PMC 2023 Sep 8. (PMC7615045; doi:10.3390/su14063280)
Supplement: Supplementary Materials [file EMS186864-supplement-Supplementary_Materials.docx]

**Supplementary Material**

**Action learning: Conceiving the interrelationships of the sustainable and healthy food systems in South Africa**

Revans’s^94^action learning conceptwas applied to capture the learning outcome of the workshop. The concept specifies that unless problems are open to a purely technical solution,there is more learning to be grasped prior to action beingtaken by those involved with an issue.It constitutes of (i)System alpha which centres on the investigation of the problem, examining the external context, structural values and available resources; (ii) System betafocuses on problem resolution, through decision cyclesof negotiation and reflection and, (iii) System gammaconcerns the participants cognitive framework, theirassumptions and prior understanding, and is concernedwith learning as experienced by each stakeholder type. The three systems, alpha, beta and gamma, are not linear or sequential, nor are they entirely discrete. All types of stakeholder possess “Programmed Knowledge’’ which can only help individuals or organisations up to a point. However, dealing with change requires greater insight and this is gained by posing ‘’Questions’’. Therefore, ‘’Learning’’ then becomes a function of acquiring programmed knowledge and combining it with questioning insight, expressed by Reg Revan’s Learning Equation:

L (Learning) = P (Programmed Knowledge) + Q (Questioning Insight)

The principal interest in developing effective learning to achieve adaptation and deal with change, was to focus on Q, Questioning Insight. It is the ability to exploit the questioning insight that would give rise to the interrelated multiple perspectivesin co-designing the SHEFS programme objectives. Action learning recognises that, in the absence of insight, the use to which anabundanceof programmed knowledge may be put is limited. Problems and opportunities are treated by leaders (in funded research these refer to programme managers/ principal investigators) who must be aware of their value systems, differing between individuals (i.e. stakeholders), and of the influences of their past personal experiences^94^.

**Supplementary Material Fig. 1**A describes the workshop process and causal loop diagramming. **A** - Systems alpha: context-specificity consideration. Systems beta: which componentsand/or lenses to consider in order to intervene optimally andfocus on theinvestigation of the problem. System gamma: Focus on the learning i.e. how to intervene collectively based on the dimensions identified. The three systems are best understood as a whole, with interlocking yet overlappingparts^95^.**B** - Unpacking the systemic interactions of the problem context through a logical framework that identify relevance with the SDGs. **C**- Developing leverage points, identified from processes in A, in order to generate evidence capable of stimulating the policy making process through alignment within a multiple level perspective (Niche-Regime-landscape) of the transition theory. Part C of the diagram is adapted from Geels^53^.
